# Two new species and one new regional record of *Indonemoura* from Guangxi, China, with additions to larval characters (Plecoptera, Nemouridae)

**DOI:** 10.3897/zookeys.825.31663

**Published:** 2019-02-18

**Authors:** Raorao Mo, Guoquan Wang, Ding Yang, Weihai Li, Dávid urányi

**Affiliations:** 1 Guangxi key laboratory of Agric-Environment and Agric-Products Safety and National Demonstration Center for Experimental Plant Science Education, Agricultural College, Guangxi University, Nanning, China Guangxi University Nanning China; 2 Department of Entomology, China Agricultural University, 2 Yuanmingyuan West Road, Beijing 100193, China Henan Institute of Science and Technology Xinxiang China; 3 Department of Plant Protection, Henan Institute of Science and Technology, Xinxiang, Henan 453003, China China Agricultural University Beijing China; 4 Plant Protection Institute, Centre for Agricultural Research, Hungarian Academy of Sciences, Herman Ottó út 15, Budapest H-1022, Hungary Centre for Agricultural Research, Hungarian Academy of Sciences Budapest Hungary; 5 Department of Zoology, Hungarian Natural History Museum, Baross u. 13, Budapest H-1088, Hungary Department of Zoology, Hungarian Natural History Museum Budapest Hungary

**Keywords:** China, *
Indonemoura
*, new record, new species, Plecoptera

## Abstract

Two new species of Nemouridae of the genus *Indonemoura* Baumann, 1975, *Indonemouraquadrata***sp. n.** and *Indonemouraquadrispina***sp. n.**, are described from Guangxi Zhuang Autonomous Region of southern China, on the basis of both sexes and larval stage. The affinities towards related species are discussed, together with generic characters of the larvae. *Indonemourascalprata* (Li & Yang, 2007) is recorded from Guangxi for the first time, and its hitherto unknown female is described.

## Introduction

The genus *Indonemoura* Baumann, 1975, a member of the Amphinemurinae, currently contains 55 species worldwide, and is mainly distributed in the Oriental Region but with a few species also in the Eastern Palaearctic (Shimizu 1994; Sivec and Stark 2010; Yang et al. 2015; Fochetti and Ceci 2016; Li et al. 2017a, b; DeWalt et al. 2018). Presently 24 species are known from China (Wu 1935, 1938, Yang and Yang 1991; Zhu et al. 2002; Li et al. 2005, 2017a, b; Li and Yang 2005, 2006, 2007, 2008a, b, c; Wang et al. 2006; Wang and Du 2009, Yang et al. 2015), and three species: *I.furcoloba* Li & Yang, 2017 (in: Li et al. 2017b), *I.voluta* Li & Yang, 2008b, and *I.yangi* Li & Yang, 2006 are known from Guangxi (Li and Yang 2006, 2008b; Li et al. 2017b). In the present paper, two new species are described from Guangxi Zhuang Autonomous Region of southern China. Additionally, *I.scalprata* (Li & Yang, 2007) is newly recorded for Guangxi.

## Materials and methods

Specimens were collected by hand or using an aerial net and are stored in 75% ethanol. The terminalia used for illustrations were cleared in 10% KOH. Types are deposited in the Department of Plant Protection, Henan Institute of Science and Technology (**HIST**) and the Collection of Smaller Insect Orders, Department of Zoology, Hungarian Natural History Museum, Budapest (**HNHM**), respectively, as indicated in the text. Illustrations were made with the aid of a Leica S8APO microscope, further colour illustrations were made with the aid of Imaging Source CCD attached to a Leica M420 microscope. The morphological terminology follows that of Baumann (1975).

## Taxonomic part

### Indonemoura quadrata sp. n.

Taxon classificationAnimaliaPlecopteraNemouridae

http://zoobank.org/2ACE6570-AA65-4336-A091-D89B13F4C6FA

**Figure 1.**
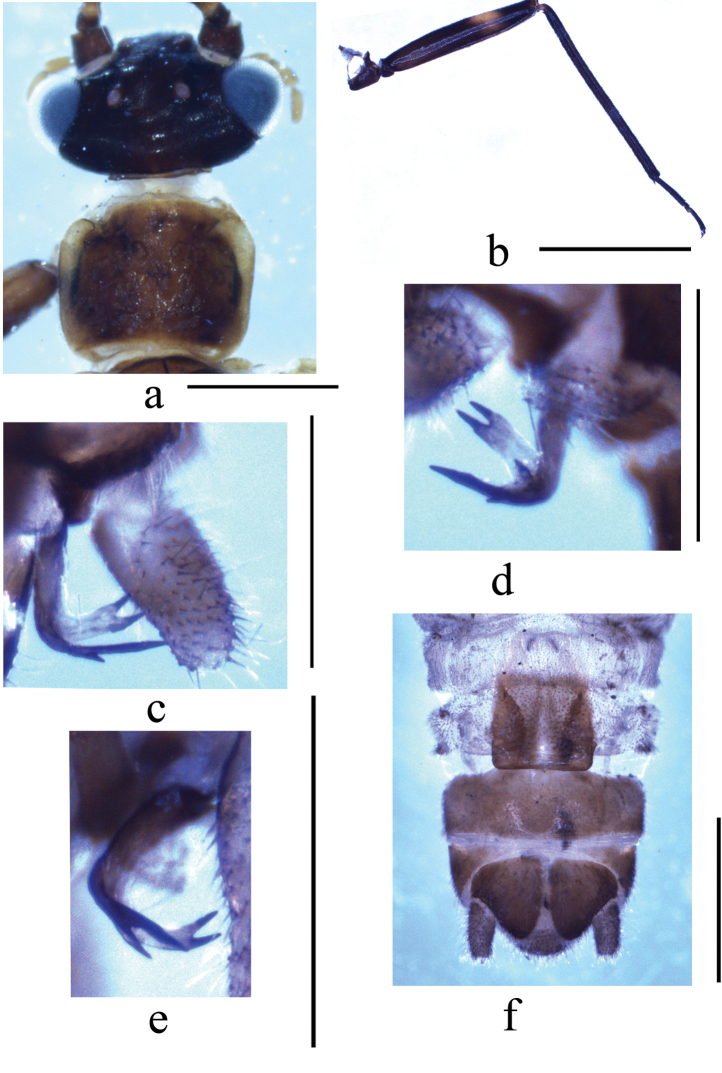
*Indonemouraquadrata* sp. n. (**a–e** male **f** female) **a** head and pronotum, dorsal view **b** hindleg, lateral view **c** left paraproct, spines of outer lobe, ventral view **d** right paraproct, spines of outer lobe, ventral view **e** right paraproct, spines of outer lobe, dorsolateral view **f** terminalia, ventral view. Scale bars: 0.5 mm.

**Figure 2.**
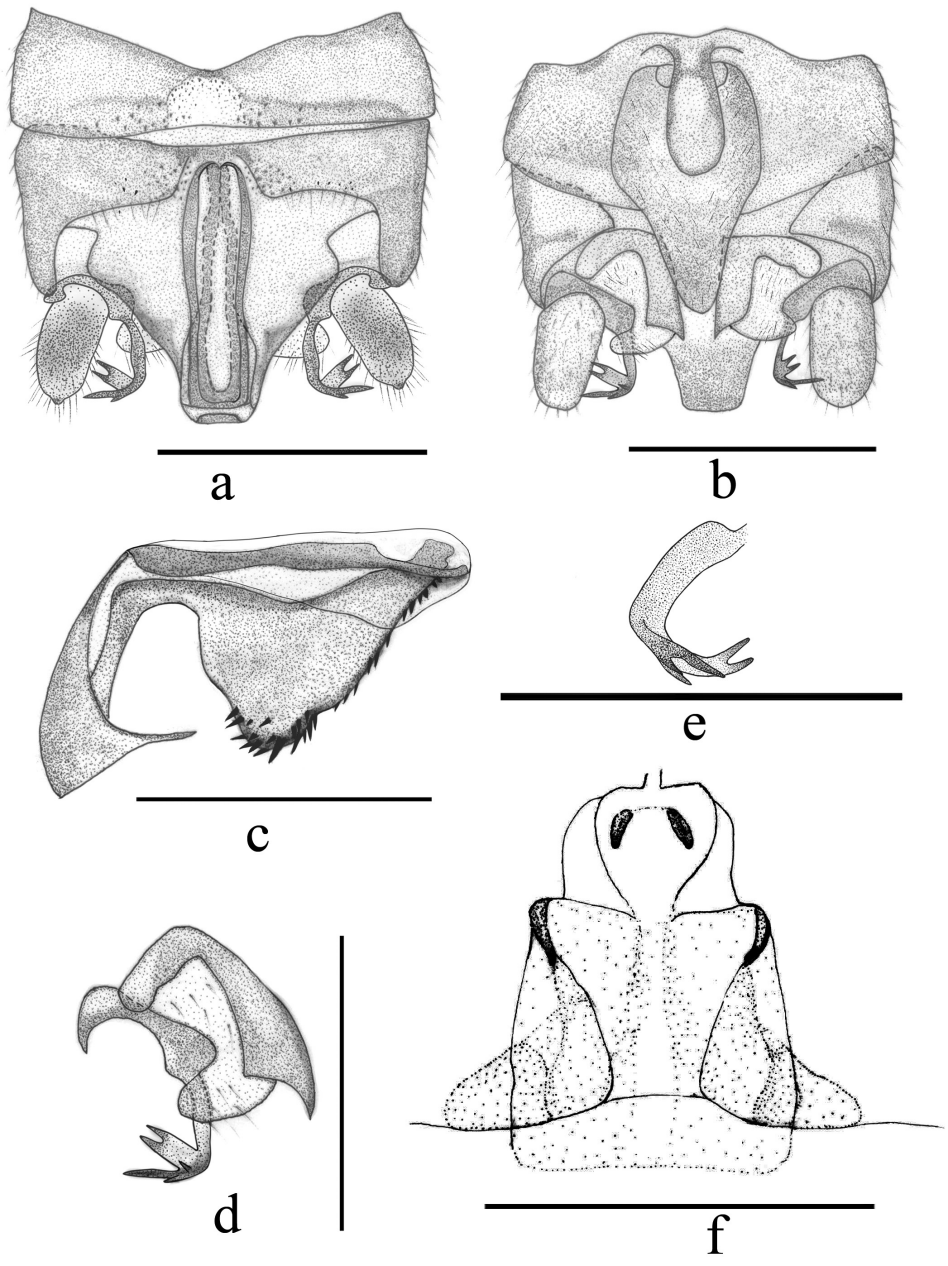
*Indonemouraquadrata* sp. n. (**a–e** male, **f** female) **a** terminalia, dorsal view **b** terminalia, ventral view **c** epiproct, lateral view **d** right paraproct, ventral view **e** right paraproct, spines of outer lobe, lateral view **f** inner genitalia, dorsal view. Scale bars: 0.5 mm.

**Figure 3.**
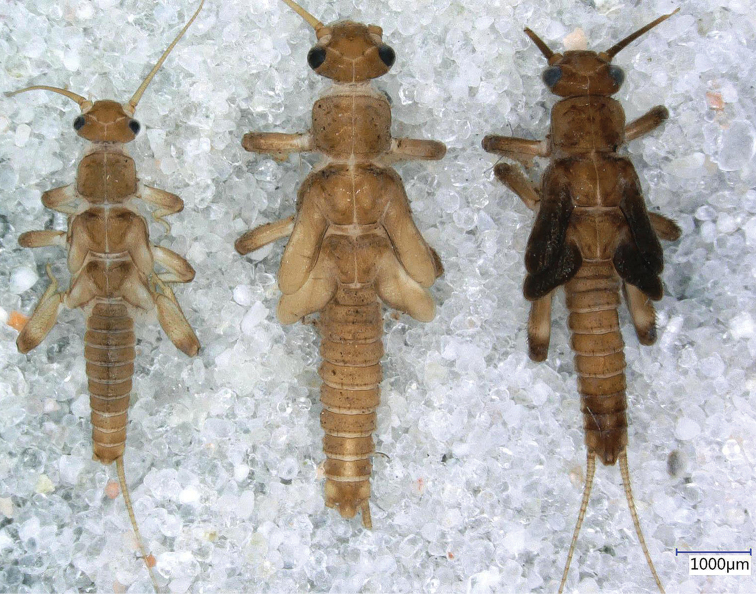
*Indonemouraquadrata* sp. n. penultimate, ultimate, and pharate male larvae (from left to right).

**Figure 4.**
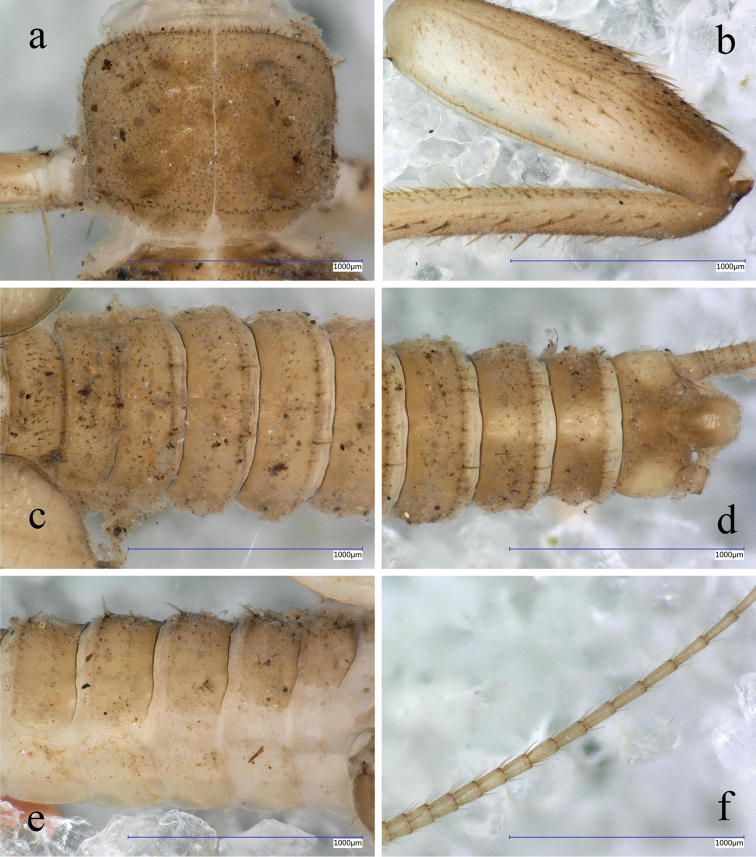
*Indonemouraquadrata* sp. n. matured larvae **a** pronotum, dorsal view **b** left hind leg, outer face **c** terga I–V , dorsal view **d** male terminalia, dorsal view **e** terga I–V , lateral view **f** cercomeres 10–22, dorsal view.

Figs 1 , 2 , 3 , 4 , 10a, b

#### Adult habitus

(Fig. 1a, b). Medium sized species, forewing length in males 6.8–7.4 mm, females 7.9 mm. Head and mouthparts dark brown, antennae brown; compound eyes black. Thorax brown; pronotum darker with pale band along lateral margins; legs (Fig. 1b) mostly dark brown, distal half of hind femora with distinct yellow brown band; wings subhyaline with darker veins; Abdominal segments mostly brown except terminalia darker; hairs on abdomen mostly pale brown.

#### Male Terminalia

(Figs 1c–e, 2a–e). Tergum IX (Fig. 2a) distinctly sclerotised, gradually constricted medially, bearing scattered, tiny black spines along mid-posterior margin; the median part of tergum IX weakly sclerotised and semicircular. Sternum IX (Fig. 2b) with claviform vesicle, slightly constricted basally; hypoproct broad and subquadrate at basal half, then gradually tapering toward tip, and covered by dense hairs. Tergum X mostly sclerotised, with narrow longitudinal concavity beneath epiproct. Cercus slightly sclerotised, nearly cylindrical with distinct hairs and a black oval process at tip, length varies ca. 2–3× width. Epiproct (Fig. 2a, c) basal half nearly parallel-sided, apical half slightly enlarged in dorsal view, with distinct apical incision; ventral sclerite strongly sclerotised, broad at base and becoming narrower toward apex, expanded ventrally into a very large semicircular ridge with rows of black spines, and the middle of ventral sclerite with two incisions forming a small semicircular process. Paraproct (Figs 1c–e, 2a, b, d, e) divided into three lobes: inner lobe sclerotised and slender, adhering to median lobe and mostly hidden by hypoproct then hardly observed; inner portion of median lobe sclerotised forming a hook-like structure with sharp tip, and remainder membranous with dense hairs at the apex; outer lobe darkly sclerotised, much longer than median lobe with two subapical prongs; the outer flat prong pale brown and curved ventrad, bifurcate apically; the inner prong much slim, forked subapically with a shorter spine ca. 1/3 of other spine.

#### Female

(Figs 1f, 2f). Sternum VII membranous, two dark inner sclerite belonging to inner genitalia easily detected by transparency. Sternum VIII with large quadrate sclerotised subgenital plate covering the entire length, posterior margin slightly concaved, slightly overlapping the anterior margin of sternum IX, paired paragenital plate is pale brown and triangular, located at the posterolateral corner of the subgenital plate, inner portion fused with subgenital plate, being dark brown and nearly semicircular which also can be seen by transparency. Sternum IX sclerotised anteriorly forming a produced arch shaped extension. Sternum X and paraproct typical. Inner genitalia mostly membranous, anterolateral margin of subgenital plate with a pair of curved thin sclerites; a large, beneath the spermathecal ductus with a pair of slender central sclerite.

#### Mature larva

(Figs 3, 4): Body relatively slender, body length without antennae and cerci 5.5–7.5 mm. General colour brown, with contrasting pale pattern on terminal segments of abdomen, less distinct dark brown pattern on thorax and distinct bands on the femora. Terminal pattern consists of paired lateral and a posteromedial light patch on tergum X (Fig. 3 penultimate and ultimate larvae), and medial light patches on terga VIII–IX; dark bands on femora occupies distal fourth. Antennae, mouth parts and cerci pale, as well as ventral aspect of the body. Setation long but less distinct. Legs moderately long, width of hind femora more than 1/3 of their length. The pronotum is trapezoidal with rounded corners, wider than long, as wide as head. Cervical gills shorter than the length of cervical sclerite where the gill is attached. Wing pads twice as long as the corresponding segments. Abdomen relatively slender, integument light matt brown, first five abdominal segments terga and sterna entirely divided by pleura, next two partly divided by pleura. Posterior margin of sternum IX of the male larva shortly rounded, sternum VIII of female larva slightly incised; paraprocts blunt. Cerci long, with 30–32 cylindrical segments; length of the 15^th^ segment is ca. two times of its width.

Setation of the larva (Fig. 4): Head, antennae and palpi with dense short setae. Pronotum covered with very short setae; marginal setae distinct and blunt, row continuous but setae in anteromedial and posteromedial half, corners have the longest setae that are as long as one 20^th^ of pronotum width (Fig. 4a). Setae on meso- and metanotum short, as long as marginal setae on pronotum; wing pads with short, acute setae. Legs with dense setation, all tibia bears indistinct swimming hairs shorter than femur width (Fig. 4b). Longest acute setae of all outer femur margins are longer than fourth of the corresponding femur width, not arranged in line but restricted to apical half. Tarsi and claws typical. Tergal segments covered with short setae of different width; row of posterior margin with distinctly longer, acute paired setae reaching ca. third of segment length, but on posterior terga more, similar long setae occur; paired setae slightly raised in lateral view (Fig. 4c–e). Cercal segments with sparse and indistinct intercalary setation but apical setae dense and relatively long; cercomeres 14–16 with an apical whorl of 10–12 acute setae that are as long as 2/3 of segment length (Fig. 4f).

#### Type material.

Holotype: 1 male (HIST), China: Guangxi Province, Wuming County, Damingshan National Natural Reserve, stream and seep beneath Golden Turtle Waterfall, 1150 m, 23°30.373'N, 108°26.141'E, 2015.III.21, leg. J Kontschán, JN Li, S Li, WH Li, D Murányi, GQ Wang. Paratypes: 2 males and 1 female (HIST), 1 male, 3 pharate male and 2 pharate female larvae, 18 penultimate and ultimate instar larvae, and 1 exuviae (HNHM), same data as holotype; Guangxi Province, Wuming County, Damingshan National Natural Reserve, inflow stream above Dragon Lake, 1225 m, 23°29.751'N, 108°26.242'E, 2015.III.22, leg. J Kontschán, JN Li, S Li, WH Li, D Murányi, GQ Wang: 2 males (one with its exuviae), 3 ultimate instar larvae, and 1 exuviae (HNHM).

#### Etymology.

The specific name refers to the sternum VIII of female with quadrate subgenital plate.

#### Distribution.

China (Guangxi).

#### Ecology.

The species was found only on the plateau of the Daming Mountains, inhabiting the same two habitats where the recently described *Cryptoperlateana* Li & Murányi, 2018 was collected (Fig. 10a, b). These two species, as well as *Rhopalopsoletriangulis* Li, Murányi & Yang, 2017 (in Li et al. 2017c) seem to be connected to the ‘tea-coloured’ waters of the Damingshan plateau. March is the beginning of its emergence period, since many penultimate and ultimate stage larvae were still in the hygropetric water layer, while only a few pharate and emerged adults were found.

#### Remarks.

*Indonemouraquadrata* is a member of the *fujianensis* complex which is characterised by ventral sclerite of epiproct with a wide, semicircular structure in lateral view. There are nine species in the *fujianensis* complex (Sivec and Stark 2010); including seven recorded from China: *I.auriformis* Li & Yang, 2008a, *I.baishanzuensis* Li & Yang, 2006, *I.fujianensis* Li & Yang, 2005, *I.guangdongensis* Li & Yang, 2006, *I.hubeiensis* Yang & Yang, 1991, *I.macrolamellata* (Wu, 1935), and *I.yangi* Li & Yang, 2006; *I.clavata* Sivec & Stark, 2010 and *I.tricantha* Sivec & Stark, 2010 have been described from Vietnam. The members of the complex can also be distinguished from the basis of the outer lobe of paraproct, that is elongated, slender, and armed with spikes.

*Indonemouraquadrata* is most similar to *I.fujianensis* Li & Yang, 2005 from Fujian both in number of spines on the outer lobes of left and right paraprocts. However, the new species may be easily separated from *I.fujianensis*: ventral sclerite of epiproct with an apical semicircular projection in lateral view; outer lobe with two prongs at apex, and both bifurcate subapically; the outer prong is flat and the size of spines approximately equal; the inner prong’s spines with different length; especially, outer lobe of right paraproct added a black spine at distal half which cannot be seen on the left paraproct. In *I.fujianensis*, apical spines of the outer lobe number three and two on the left and right paraprocts respectively (Li and Yang 2005: figs 2, 5). This species also easily confused with *I.auriformis* by the dorsal view of terminalia because of the same inner prong of the outer paraproct lobe, especially when the outer prong of the outer lobe is hardly observed sometimes due to its paler colour and more or less erect position (Li and Yang 2008a: figs 8, 11). Among the congeners where females are known, the female of *I.quadrata* is distinctive by its regular quadrate subgenital plate. Hitherto only two congeners, the Himalayan *I.adunca* (Harper, 1974) and *I.indica* (Kimmins, 1947) are known in the larval stage (Sivec 1981). On the basis of these two Himalayan species, the two species described herein and a further larva that we recently reported from Shaanxi as *Indonemoura* sp. (Li et al. 2018), there are no distinctive generic character to distinguish *Indonemoura* larvae from those of *Mesonemoura* Baumann, 1975. Both genus can be characterised by the presence of single, short cervical gills, posterior row of setae on terga having longer paired setae, and long setae on femora not arranged in line. The larva of *I.quadrata* can be distinguished from the few known congeners on the basis of characteristic pale and dark pattern on terga VIII–X. However, similar pattern was observed on the Shaanxi larvae, suggesting the pattern is not a specific character.

### Indonemoura quadrispina sp. n.

Taxon classificationAnimaliaPlecopteraNemouridae

http://zoobank.org/1C0796EA-5B4D-4127-9EDF-969693F01BEE

**Figure 5.**
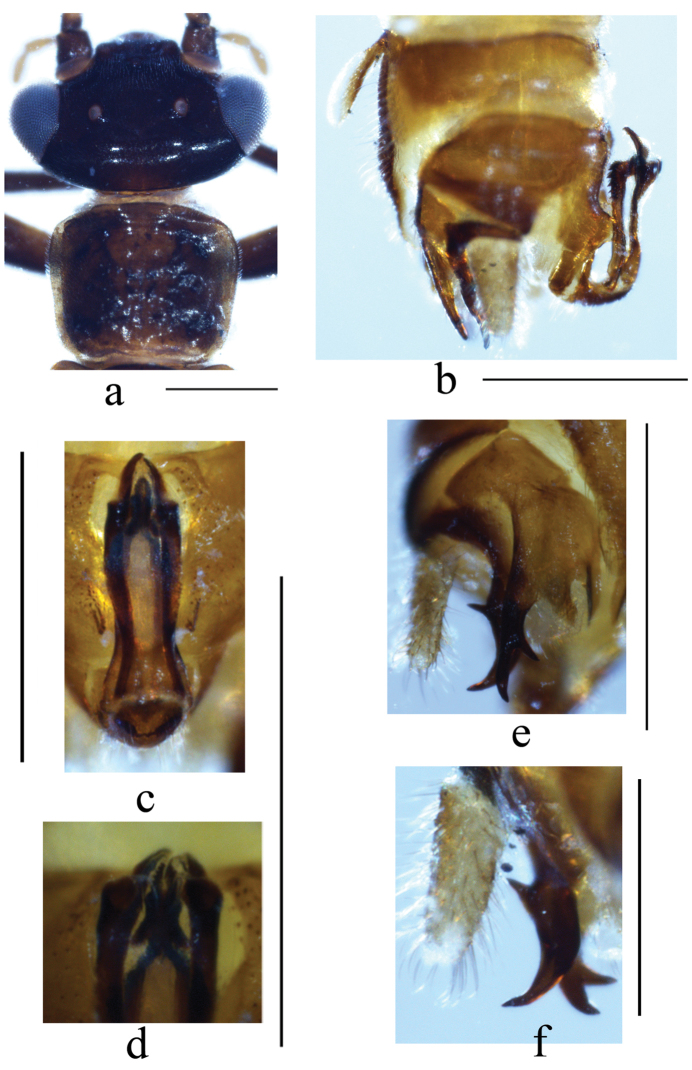
*Indonemouraquadrispina* sp. n. (male) **a** head and pronotum, dorsal view **b** terminalia, lateral view **c** epiproct, dorsal view **d** apex of epiproct, caudal view **e** right paraproct, ventral view **f** apex of mesal and outer left paraproct lobes, dorsal view. Scale bars: 0.5 mm.

**Figure 6.**
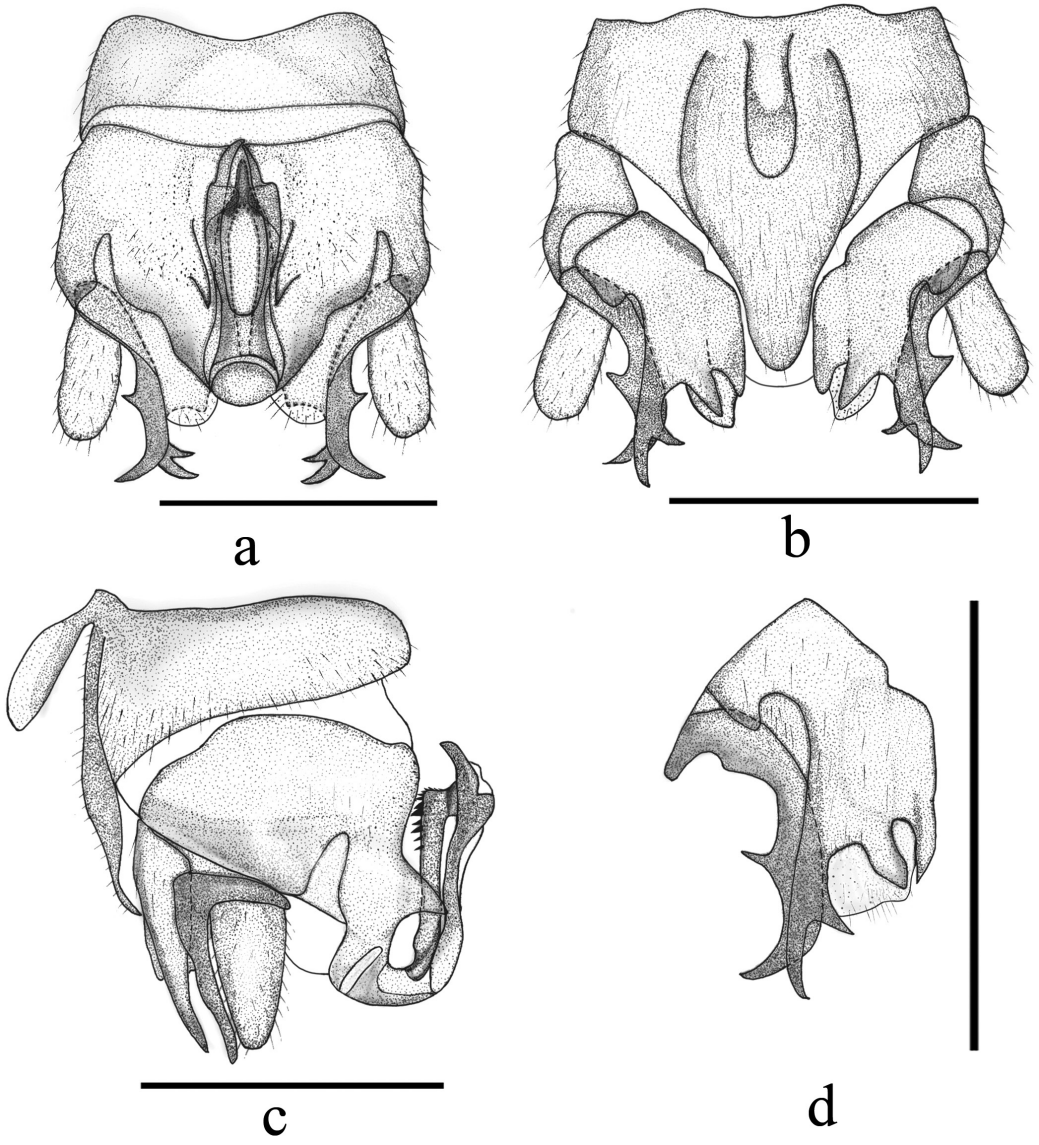
*Indonemouraquadrispina* sp. n. (male) **a** terminalia, dorsal view **b** terminalia, ventral view **c** terminalia, lateral view **d** right paraproct, ventral view. Scale bars: 0.5 mm.

**Figure 7.**
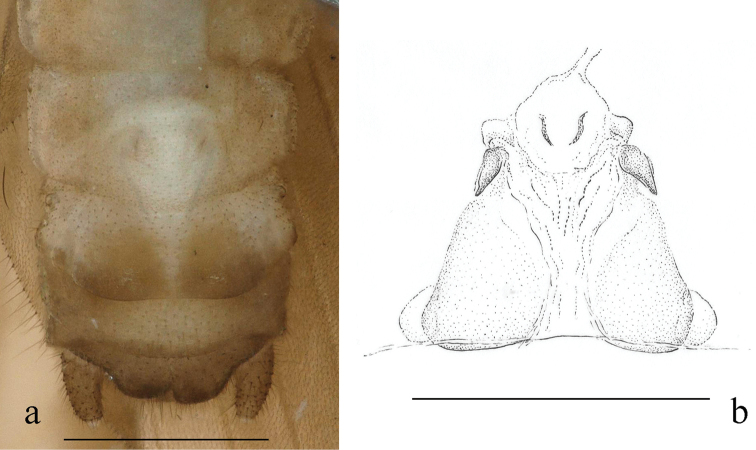
*Indonemouraquadrispina* sp. n. (female) **a** terminalia, ventral view **b** inner genitalia, dorsal view. Scale bars: 0.5 mm.

**Figure 8.**
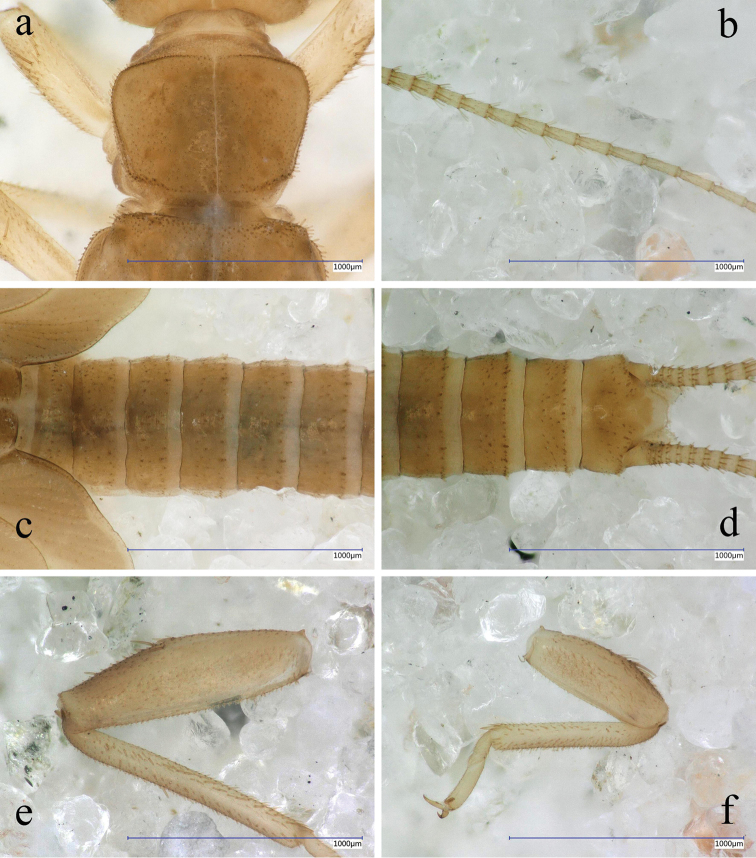
*Indonemouraquadrispina* sp. n. matured male larva **a** pronotum, dorsal view **b** cercomeres 10–21, dorsal view **c** terga I–VI , dorsal view **d** terminalia, dorsal view **e** right hind leg, outer face **f** right foreleg, outer face.

Figs 5 , 6 , 7 , 8 , 10c

Indonemoura sp. n.: Gamboa et al. 2019: S1 Table, using the sequence of the present specimens in phylogeny analysis.

#### Adult habitus

(Fig. 5a). Medium sized species, forewing length in males 5.6–6.2 mm, females 6.5 mm. General colour brown. Head, mouth parts and legs dark brown; antennae brown; compound eyes dark. Thorax including pronotum dark brown; pronotum (Fig. 5a) nearly trapezoidal, corners bluntly round, lateral margins light brown, midlateral portion with dark rugosity. Wings brownish, subhyaline with dark veins. Abdominal segments brownish but terminalia darker with hairs light brown.

#### Male terminalia.

Tergum IX (Fig. 6a) distinctly sclerotised anteriorly, with a large triangular mid-posterior incision. Sternum IX (Fig. 6b, c) with claviform vesicle; vesicle mostly membranous except anterior and lateral margins sclerotised, with an oval mid-anterior membrane, length greater than 4× width; hypoproct broad and nearly rectangular at basal half, then gradually tapering to a nipple-like tip. Tergum X distinctly sclerotised, with a deep median concavity present beneath epiproct, and a pair of upraised triangular process present mesolaterally. Cercus slightly sclerotised and nearly cylindrical, with many clothing hairs, length ca. 2.5× width. Epiproct (Figs 5b–d, 6a, c) recurved and long; dorsal sclerite nearly gourd-shaped and basal half roughly circular, slightly constricted medially, rectangular subapically, then distinctly tapering toward sharp tip; ventral sclerite strongly sclerotised, broad at base and becoming narrower toward apex, expanded ventrally into a straight ridge with rows of spines before fusing with dorsal sclerite; bearing a pair of dark thorn-like subapical structures. Paraproct (Figs 5b, e, f, 6a–d) divided into three lobes: inner lobe sclerotised, slender and rectangular basally, with an acute tip; median lobe mostly sclerotised and broad at base with a large triangular distal denticle at inner portion and a large, forked spine subapically, distinctly curved inward apically at outer margin; outer lobe strongly sclerotised, horn-shaped with a small projection near mid-point.

#### Female

(Fig. 7). Sternum VII membranous, with two dark markings at its inner and median portion, as inner genitalia structures appear by transparency. Sternum VIII with wide, bicolored subgenital plate covering two third of length and slightly overhanging, its width is more than two thirds of segment’s width; paired paragenital plate small and rounded, indistinct, attached to posterolateral sides of the subgenital plate. Lateral portions of the subgenital plate dark brown, medial area light and posteriorly narrowing; posterior margin laterally rounded and medially slightly concave. Sternum IX lightly sclerotised, anterior margin overlapped by the subgenital plate. Sternum X and paraproct typical. Inner genitalia mostly membranous, with two small lateral sclerites attached to the anterolateral edges of the subgenital plate, and a large, bulbous structure beneath spermathecal ductus, involving a pair of small central sclerite; genital opening narrow.

#### Mature larva

(Fig. 8): Body relatively slender, body length without antennae and cerci 4.8–5.2 mm. General colour brown, with indistinct dark brown pattern on thorax and slightly darker apical third on femora. Antennae, mouth parts and cerci pale, as well as ventral aspect of the body. Setation short and indistinct on the body but legs and cerci armed with longer setae. Legs moderately long, width of hind femora more than 1/3 of their length. The pronotum is trapezoidal with rounded corners, wider than long, as wide as head. Cervical gills shorter than the length of cervical sclerite where the gill is attached. Wing pads twice as long as the corresponding segments. Abdomen slender, integument yellowish matt brown, first six abdominal segments completely, tergum VII partly divided by pleura. Posterior margin of sternum IX of the male larva sharply triangular in the middle, sternum VIII of female larva slightly incised; paraprocts slightly elongated but apex blunt. Cerci long, with 29–32 cylindrical segments; length of the 15^th^ segment is ca. two times of its width.

Setation of the larva (Fig. 8): Head, antennae, and palpi with moderately dense, short setae. Pronotum covered with very short and scarce setae; marginal setae very short and blunt, of which bases are narrower than the rounded apices, row interrupted in anteromedial and posteromedial fifth, corners have the longest setae that are as long as one 35^th^ of pronotum width (Fig. 8a). Setae on meso- and metanotum slightly longer than marginal setae on pronotum; wing pads with very short, blunt setae. Legs with scarce setation, all tibia bears indistinct, scarce swimming hairs as long as half of femur width (Fig. 8e, f). Longest acute setae on fore femora as long as half of femur width, while on hind femora only as one third of femur width; long setae not arranged in line, restricted to mediodorsal area. Tarsi and claws relatively long. Tergal segments covered with very short setae; row of posterior margin with slightly longer, blunt paired setae reaching ca. one seventh of segment length, but on posterior terga more, similar long setae occur (Fig. 8c, d). Cercal segments with sparse but relatively long intercalary setation, apical setae dense and relatively long; cercomeres 14–16 with an apical whorl of 9–11 acute setae that are longer than half of segment length (Fig. 8b).

#### Type material.

Holotype: 1 male (HIST), China: Guangxi Zhuang Autonomous Region, Shangsi County, Shiwandashan National Natural Reserve, forest seep by the Pearl River, 265 m, 21°54.216'N, 107°54.240'E, 2015.Ⅲ.27–29, leg. J Kontschán, JN Li, S Li, WH Li, D Murányi, GQ Wang. Paratypes: 2 males and 1 female, 2 pharate male larvae, 3 ultimate instar larvae (HNHM), same data as holotype.

#### Etymology.

The specific name is a noun in apposition, referring to four apical spines on the median lobe and outer lobe of the paraproct.

#### Distribution.

China (Guangxi).

#### Ecology.

The species was found only in a small forest seep by the Pearl River in the Shiwandashan National Natural Reserve (Fig. 10c). The seep is having sandy or fine gravel substrate with plenty of organic materials like fallen leaves. Besides the new species, adults of *Rhopalopsolecestroidea* Li, Murányi & Gamboa, 2017 (in: Li et al. 2017c), *Amphinemurahainana* Li & Yang, 2008d and *A.hamiornata* Li & Yang, 2008c were also collected beaten from the riparian plants around the seep. However, only larvae of the new species were found in the water, and other adult stoneflies probably emerged from the Pearl River flowing only a few meters far from the seep. March seems to be the main season of its emergence, since more adults than larvae were found.

#### Remarks.

The new species seems to be most similar to *I.curvicornia* Wang & Du, 2009 described from Zhejiang in the dorsal aspect of the long and narrow epiproct and similar outer lobe of paraprocts, especially the horn-shape of outer lobe with a small projection at apical half. However, the new species may be separated from *I.curvicornia* by its dorsal sclerite gourd-shaped in dorsal view and ventral sclerite of epiproct with straight ridge in lateral aspect; median lobe with a subtriangular denticle at apical inner portion and its outer portion is forked subapically. In *I.curvicornia*, the ventral sclerite of slim epiproct with narrow keel; median lobe membranous at inner portion and its outer portion slim and unforked apically (figs 15–19, 21 in Wang and Du 2009). Similar bifurcate median paraproctal lobes also occur in *I.trichotoma* Li & Yang, 2008b from Yunnan Province, but it is easily separated from that species by the forked outer paraproctal lobe and the spineless hypoproctal apex (comparing Figs 6–7 in Li and Yang 2008b and Figs 3b, e, f, 4a–d). The female of *I.quadrispina* is less distinctive, simple subgenital plate is similar to several congeners, e.g *I.clavata* Sivec & Stark, 2010 or the *I.scalprata* female described below. It can be distinguished from the latter on the basis of bicolored, wider, and less concave subgenital plate. The larva can be easily distinguished from known larvae of congeners on the basis of its very short tergal setation.

### Indonemoura scalprata

Taxon classificationAnimaliaPlecopteraNemouridae

(Li & Yang, 2007)

Figs 9 , 10d

Amphinemura scalprata : Li and Yang 2007: 61 (original description of the male from Guangdong).
Indonemoura scalprata : Yang et al. 2015: 284 (comb. n. and first record from Fujian).
Indonemoura scalprata : Gamboa et al. 2019: S1 Table, using the sequence of the present specimens in phylogeny analysis.

#### Description of the female

**Figure 9.**
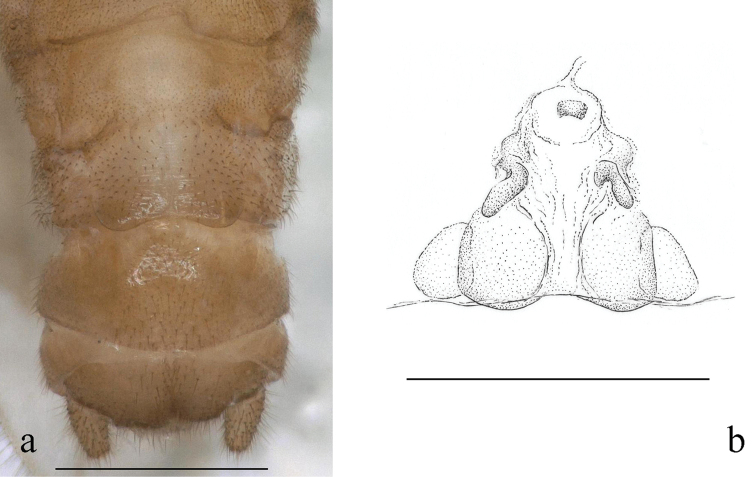
Indonemourascalprata (Li & Yang, 2007) (female) **a** terminalia, ventral view **b** inner genitalia, dorsal view. Scale bars: 0.5 mm.

(Fig. 9). Forewing length 6.5–7.2 mm; coloration similar to male. Sternum VII membranous, lacks pregenital plate. Sternum VIII with wide, entirely brown subgenital plate covering more than two third of length and slightly overhanging, its width is less than two thirds of segment’s width; paired paragenital plate relatively large but indistinct, rounded triangular, weakly attached to lateral sides of the subgenital plate. Posterior margin of the subgenital plate laterally rounded and medially slightly concave. Sternum IX lightly sclerotised, anterior margin overlapped by the subgenital plate. Sternum X and paraproct typical. Inner genitalia mostly membranous, with two small lateral sclerites attached to the anterolateral edges of the subgenital plate, and a medium sized, bulbous structure beneath spermathecal ductus, involving a central sclerite ring; genital opening narrow.

#### Material examined.

China: Guangxi Zhuang Autonomous Region, Wuming County, Liangjiang Town, Neichao, Neichao River above Neichao Ming Hotel, 220 m, 23°29.664'N, 108°21.622'E, 2015.VII.24, leg. JN Li, S Li, WH Li, D Murányi: 1 male and 3 females (HNHM); the same locality, 230 m, 23°29.457'N, 108°21.600'E, 2017.Ⅶ.13, leg. RR Mo, Y Lai: 1 male (HIST); China: Guangxi, Laibin City, Jinxiu County, Dayaoshan National Nature Reserve, Yinshan Park, 1210 m, 24°16.133'N, 110°36.924'E, 2012.IV.8. Leg. WH Li: 1 male (HIST).

#### Distribution.

China (Fujian, Guangdong, and Guangxi).

#### Ecology.

**Figure 10.**
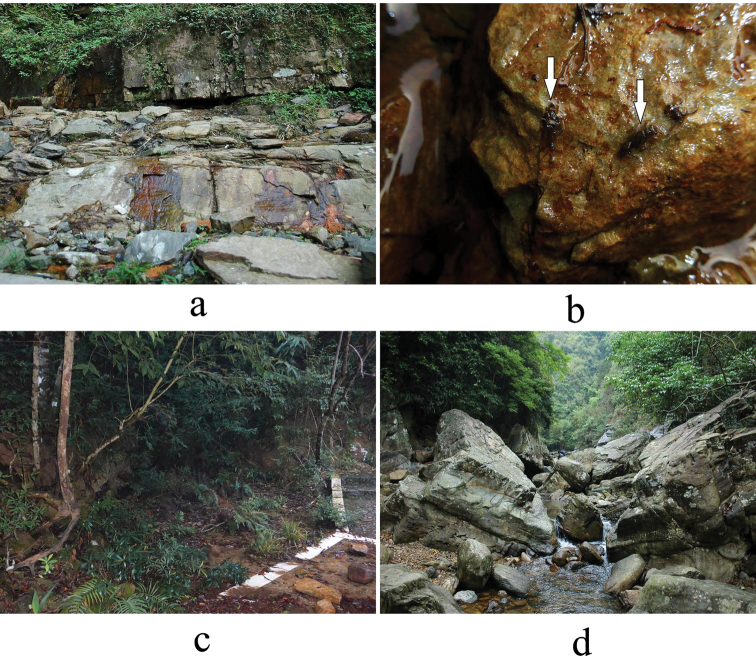
Habitats of Indonemoura from Guangxi **a** type locality of I.quadrata sp. n., Damingshan N.N.R. **b** exuviae (left arrow) and larva (right arrow) of I.quadrata sp. n. in the hygropetric water at the type locality **c** type locality of I.quadrispina sp. n., Shiwandashan NNR **d** habitat of I.scalprata (Li & Yang, 2007), Neichao.

In Guangxi, the species was found in a large, rocky stream by the foothills of the Damingshan (Fig. 10d). Other adult stoneflies collected at the locality are Rhopalopsolesinensis Yang & Yang, 1993 and a yet undescribed Amphinemura sp. Not any Euholognatha larvae were found, but several larvae and exuviae of Perlidae (Agnetina sp., Neoperla sp., Togoperla sp.). All the I.scalprata specimens were fully coloured, indicating that emergence period was past its peak. 

#### Remarks.

The female of I.scalprata is less distinctive, similar to the female of I.quadrispina as described above.

## Concluding remarks

Previous studies on the genus *Indonemoura* from Guangxi were explained by Li and Yang, who described *I.voluta* from Mountain Mao’ershan of Guilin (Li and Yang 2008b) and *I.yangi* from Mountain Jiuwandashan of Huanjiang (Li and Yang 2006). Recently, one new species *I.furcoloba* was added from Mountain Shengtang in Jinxiu by Li et al. (2017b). There was no record of this genus in previous works on the insect fauna of the Damingshan National Natural Reserve and Shiwandashan National Natural Reserve (Du and Sivec 2004). In this study, two additional new species are described and one new record for Guangxi is recorded from these two ranges. Therefore, there are up to six known *Indonemoura* species from Guangxi. The five species that were described from Guangxi are not yet known out of the region, while *I.scalprata* was originally described from the bordering Guangdong Province, and is also found in Fujian Province (Li and Yang 2007, Yang et al. 2015).

## Supplementary Material

XML Treatment for Indonemoura quadrata

XML Treatment for Indonemoura quadrispina

XML Treatment for Indonemoura scalprata

## References

[B1] Baumann R-W (1975) Revision of the stonefly family Nemouridae (Plecoptera): A study of the world fauna at the generic level. Smithsonian Contributions to Zoology 211: 1–74. 10.5479/si.00810282.211

[B2] DeWalt R-E Maehr M-D Neu-Becker U Stueber G (2018) Plecoptera Species File Online. Version 5.0/5.0. http://Plecoptera.Species.File.org [Accessed 20 Jul 2018]

[B3] Du Y-Z Sivec I (2004) Plecoptera: Perlidae, Nemouridae, Leuctridae. In: Yang XK (Ed.) Insects from Mt.Shiwandashan area of Guangxi. China Forestry Publishing House, Beijing, 39–45.

[B4] Fochetti R Ceci M (2016) *Indonemouraannamensis*–a new species of stonefly from Vietnam (Plecoptera: Nemouridae). Zootaxa 4121(1): 85–88. 10.11646/zootaxa.4121.1.8 27395208

[B5] Gamboa M Murányi D Kanmori S Watanabe K (2019) Molecular phylogeny and diversification timing of the Nemouridae family (Insecta, Plecoptera) in the Japanese Archipelago. PloS ONE 14(1): e0210269. 10.1371/journal.pone.0210269 PMC6329508 30633758

[B6] Harper P-P (1974) New *Protonemura* (s.l.) from Nepal (Plecoptera; Nemouridae). Psyche 81(3–4): 367–376. 10.1155/1974/57825

[B7] Kimmins D-E (1947) New species of Himalayan Plecoptera. The Annals and Magazine of Natural History 11(13): 721–740.

[B8] Li W-H Murányi D (2018) A new species of *Cryptoperla* Needham, 1909 (Plecoptera: Peltoperlidae) from Guangxi of China, based on male, female, and larval stage. Zootaxa 4455(1): 177–188. 10.11646/zootaxa.4455.1.8 30314226

[B9] Li W-H Yang D (2005) Two new species of *Indonemoura* (Plecoptera: Nemouridae) from Fujian, China. Zootaxa 1001: 59–63. 10.11646/zootaxa.1001.1.4

[B10] Li W-H Yang D (2006) The genus *Indonemoura* Baumann, 1975 (Plecoptera: Nemouridae) from China. Zootaxa 1283: 47–61.

[B11] Li W-H Yang D (2007) Review of the genus *Amphinemura* (Plecoptera: Nemouridae) from Guangdong, China. Zootaxa 1511: 55–64. 10.11646/zootaxa.1511.1.4

[B12] Li W-H Yang D (2008a) Two new species and two new records of stonefly family Nemouridae from Henan (Plecoptera: Nemouroidea). In: Shen X Lu C (Eds) The Fauna and Taxonomy of Insects in Henan 6.China Agricultural Science and Technology Press, Beijing, 11–16.

[B13] Li W-H Yang D (2008b) Two new species of *Indonemoura* (Plecoptera: Nemouridae) from China, with redescription of *Indonemouralongiplatta* (Wu, 1949), comb. n. Aquatic Insects 30: 97–103. 10.1080/01650420701882988

[B14] Li W-H Yang D (2008c) New species of Nemouridae (Plecoptera) from China. Aquatic Insects 30: 205–221. 10.1080/01650420802334038

[B15] Li W-H Yang D (2008d) A new species of *Amphinemura* (Plecoptera: Nemouridae) from China. Zootaxa 1892: 65–68. 10.11646/zootaxa.5194.3.7 37045322

[B16] Li W-H Wu L-M Yang D (2017a) Two new species of *Indonemoura* (Plecoptera: Nemouridae) from Yunnan Province of southwestern China. Zootaxa 4231: 289–295. 10.11646/zootaxa.4231.2.11 28187545

[B17] Li W-H Yang D Sivec I (2005) Two new species of *Indonemoura* (Plecoptera: Nemouridae) from China. Zootaxa 893: 1–5. 10.11646/zootaxa.893.1.1

[B18] Li W-H Zhang Q Yang D Yao G (2017b) A new Chinese species of *Indonemoura* (Plecoptera: Nemouridae) and a new subspecies of *I.nigrihamita* Li & Yang. Zootaxa 4311: 255–262. 10.11646/zootaxa.4311.2.6

[B19] Li W-H Mo R-R Dong W-B Yang D Murányi D (2018) Two new species of *Amphinemura* (Plecoptera: Nemouridae) from south Qinling Mountains of China based on male, female and larvae. ZooKeys 808: 1–21. 10.3897/zookeys.808.29433 PMC6305769 30598606

[B20] Li W-H Murányi D Gamboa M Yang D Watanabe K (2017c) New species and records of Leuctridae (Plecoptera) from Guangxi, China, on the basis of morphological and molecular data, with emphasis on *Rhopalopsole*. Zootaxa 4243(1): 165–176. 10.11646/zootaxa.4243.1.8 28610177

[B21] Shimizu T (1994) *Indonemouranohirae* (Okamoto, 1922), comb. nov. (Plecoptera, Nemouridae) newly recorded from Japan, with a redescription of *Amphinemuralongispina* (Okamoto, 1922). Japanese Journal of Entomology 62: 619–627.

[B22] Sivec I (1981) Some notes about Nemouridae larvae (Plecoptera) from Nepal. Entomologica Basiliensia 6: 108–119.

[B23] Sivec I Stark B-P (2010) Eleven new species of the genus *Indonemoura* Baumann (Plecoptera: Nemouridae) from Thailand and Vietnam. Illiesia 6(14): 210–226.

[B24] Wang Z-J Du Y-Z (2009) Four new species of the genus *Indonemoura* (Plecoptera: Nemouridae) from China. Zootaxa 1976: 56–62.

[B25] Wang Z-J Du Y-Z Sivec I Li Z-Z (2006) Records and descriptions of some Nemouridae species (Order: Plecoptera) from Leigong Mountain, Guizhou province, China. Illiesia 2: 50–56.

[B26] Wu C-F (1935) New species of stoneflies from East and South China. Bulletin of the Peking Society of Natural History 9: 227–243.

[B27] Wu C-F (1938) *Plecopterorumsinensium*: A monograph of stoneflies of China (Order Plecoptera). Yenching University, 225 pp.

[B28] Yang D Yang C-K (1991) New species of Plecoptera from Hubei. Journal of Hubei University (Natural Science) 13: 369–372.

[B29] Yang D Yang C-K (1993) New and little-known species of Plecoptera from Guizhou Province (III). Entomotaxonomia 15(4): 235–238.

[B30] Yang D Li W-H Zhu F (2015) Fauna Sinica, Insecta Vol. 58, Plecoptera: Nemouroidea. Science Press, Beijing, 518 pp.

[B31] Zhu F Yang D Yang C-K (2002) A new species of the genus *Indonemoura* Baumann (Plecoptera: Nemouridae) from Tibet. China Entomological Science 5: 317–320.

